# Active Control of Charge Density Waves at Degenerate Semiconductor Interfaces

**DOI:** 10.1038/s41598-017-11005-6

**Published:** 2017-09-07

**Authors:** Raj K. Vinnakota, Dentcho A. Genov

**Affiliations:** College of Engineering and Science, Louisiana Tech University, Ruston, USA

## Abstract

We present an optoelectronic switch for functional plasmonic circuits based on active control of Surface Plasmon Polaritons (SPPs) at degenerate PN^+^-junction interfaces. Self-consistent multi-physics simulations of the electromagnetic, thermal and IV characteristics of the device have been performed. The lattice matched Indium Gallium Arsenide (In_0.53_Ga_0.47_As) is identified as a better semiconductor material compared to Si for the practical implementation of the proposed optoelectronic switch providing higher optical confinement, reduced size and faster operation. The optimal device is shown to operate at signal modulation surpassing −100 dB, responsivity in excess of −600 dB·V^−1^ and switching rates up to 50 GHz, thus potentially providing a new pathway toward bridging the gap between electronic and photonic devices.

## Introduction

Researchers have long realized that photonics could be a key technology for fast data communication and computing^1–5^. It is now well understood that photonic devices have potential to address some of the present bottlenecks in semiconductor based electronics such as high power consumption and interconnects delay times. Unfortunately the implementation of photonic devices within electronic components has been limited predominantly due to size mismatch, i.e. the optical components are diffraction limited to half the wavelength in the optical material. A possible solution which can facilitate the size and power requirement for future integrated circuits lies in designing photonics components below the diffraction limit.

Due to the dramatic advances in nanotechnology and its applications in the area of photonics, and specifically plasmonics, now it is believed feasible to merge electronics with sub-wavelength optics in a new field of sub-wavelength optoelectronics^1–3^. Semiconductor electronics is currently limited in speed, by heat generation and interconnect delay time^4^. Photonic devices, on the other hand, can operate at low transmission losses and provide extremely large bandwidths due to multiplexing capabilities, operating on several channels in parallel. However, dielectric waveguides and interconnects are limited by the fundamental law of diffraction and require a fast all-optical switching mechanism. Alternatively, Surface Plasmon Polaritons (SPPs), i.e. spatially confined electromagnetic modes propagating at the metal- dielectric interfaces, offer the bandwidths of photonic devices and physical dimensions shared with nanoscale electronics^1–20^.

One of the most fascinating aspects of SPPs is the way light can be channeled using device geometries much smaller than the free space wavelength. The SPPs propagate at the interface of metals/semiconductors and has been used in wide range of disciplines including bio-sensing^21^, super lensing^22–24^, nanolasing^25, 26^, optical invisibility and metamaterials^27, 28^. Active electronically controlled plasmonic element has been demonstrated as a selective ring resonator switch^29^ where the SPPs are controlled by induced small refractive index changes of order 10^−3^. A sub-micron bidirectional all-optical plasmonic switch with asymmetric T-shape single slit was recently demonstrated with transmission modulation of −6 dB^30^. All-optical absorption and gain assisted switching was demonstrated using SPP waveguide coupled with PMMA films^9^ and a cavity filled with a semiconductor (InGaAsP) gain material^31^. A metal-oxide-Si field effect plasmonic modulators and all-optical modulation by plasmonic excitation of CdSe quantum dots have been investigated at visible and telecommunication frequencies^8, 32^. Recently, a fast all-optical switch based on a carbon nanotube metamaterial has been proposed, however the device shows a rather low transmission modulation of less than 10%^33^. The SPP modulation rates so far demonstrated range from a few kHz^8^, to tens of MHz^34–36^. Overall, the rapid progress in the past few years have shown that; (*i*) exceedingly fast (tens of Gbit/s) optoelectronic switching can be achieved using dielectric components, however the device sizes are large (<100 *μm* ^2^) and the signal modulation is relatively low (few dB), while (*ii*) metal-based plasmonic modulators can have small sizes (<1 *μm* ^2^), however an efficient switching mechanism is still to be identified. Despite the progress, it is arguable that in order for optoelectronic devices to compete with their all-electronic counterparts a signal modulation surpassing −10dB and bandwidths beyond  5GHz must be achieved.

In this article, we build upon our original work on a fast optoelectronic switch termed Surface Plasmon Polariton Diode (SPPD)^37^. Specifically, we advance a new comprehensive multi-physics study of the complex phenomena behind the SPPD operation. A numerical framework is developed which self-consistently solves the Maxwell’s, Poisson-Boltzmann, drift-diffusion and heat equations. This model allows for accurate simulations of the excitation and electro-optical control of the SPPs, the minority carrier transport across the *PN* ^+^- junction, the spatially and time dependent local permittivity variations under external bias, and introduction of thermal effects due to Ohmic heating and electromagnetic energy dissipation. Combined with the use of two Figures of Merits (FOMs) our studies have identified lattice matched Indium Gallium Arsenide (In_0.53_Ga_0.47_As) as one of the best semiconductor material which offers a SPPD operation with low loss, high tunability and extreme mode confinement which could potentially lead to smaller and low dissipation optoelectronic devices with high signal modulation. In this study we also consider the constrains imposed by the present micro- and nano- manufacturing technology, by using realistic doping concentrations and electromagnetic frequency range of operation that is accessible through experimentation, specifically using CO_2_ or quantum cascade lasers (QCL)^38, 39^.

**Figure 1:**
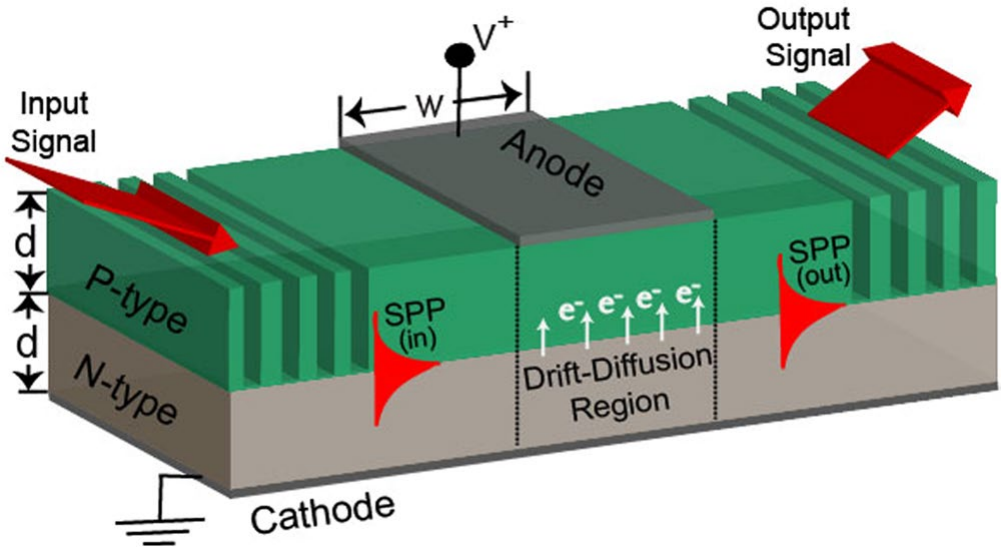
Basic schematic of a Surface Plasmon Polarition Diode (SPPD).

Basic schematic of the SPPD is shown in Fig. 1. It consists of a *PN* ^+^ - junction made of highly doped (degenerate) semiconductor with an active drift-diffusion region formed between two control electrodes. When a forward bias is applied across the device, minority carriers (electrons) are injected in the *P*- doped layer altering it dielectric constant $\,{{\epsilon }}_{p}$. For applied voltage higher than a critical value *V* > *V*_*c*_ the *P*- layer acquires a metal like characteristics impeding the propagation of the SPP across the active region and establishing the OFF state of the device. The critical voltage for a given operational frequency, *ω*_*o*_, is obtained from the transparency condition^37^ ${\rm{Re}}[{{\epsilon }}_{p}]=0$, resulting in ${V}_{c}=\frac{{k}_{B}T}{q}\,\mathrm{ln}[{\varepsilon }_{b}{\omega }_{o}^{2}/{\omega }_{p0}^{2}]$ where ${\omega }_{p0}=q{n}_{i}(T)/\sqrt{{\varepsilon }_{b}{m}_{e}{N}_{A}}$ is the renormalized plasma frequency of the minority carriers under thermal equilibrium, *m*_*e*_ is the electrons effective mass in the *P*- layer, *ε*_*b*_ is the contribution of the lattice electrons to the semiconductor permittivity, and *N*_*A*_ is the acceptor doping concentration. In all calculations that follows, the SPPD geometric characteristics are fixed with the *P*- layer having thickness *d* = 1.5 *μm* and the overall length of the active drift-diffusion region is *w* = 4 *μm*.

The operation of the SPPD depends on large set of parameters including donor and acceptor doping, temperature, applied bias, semiconductor materials and device geometry. In this study we focus on silicon (Si) and lattice matched Indium Gallium Arsenide (In_0.53_Ga_0.47_As) based devices. These two semiconductors are excellent candidates due to well-established manufacturing protocols at high (degenerate) doping levels and as shown next a superior SPP characteristics. The SPP are charge density waves that are exponentially confined at the metal/semiconductor interfaces^40–42^, and have wavelengths substantially shorter compared to those in the adjacent dielectric medium. However, with the increased confinement/localization there is a corresponding increase in propagation losses, thus for a practical applications a positive trade-off must be achieved between these two characteristics. In order to address this issue and provide a roadmap toward practically feasible SPPD we introduce two figures of merit (FOM) to quantify the SPP localization and propagation characteristics

1 $$\begin{array}{c}{{\rm{FOM}}}_{1}=|\frac{Re[{k}_{SPP}(\omega ,{N}_{D},{N}_{A})]}{Re[{k}_{P}(\omega ,{N}_{A})]}|\\ {{\rm{FOM}}}_{2}=|\frac{Re[{k}_{SPP}(\omega ,{N}_{D},{N}_{A})]-Re[{k}_{P}(\omega ,{N}_{A})]}{Im[{k}_{SPP}(\omega ,{N}_{D},{N}_{A})}|.\end{array}$$

**Figure 2:**
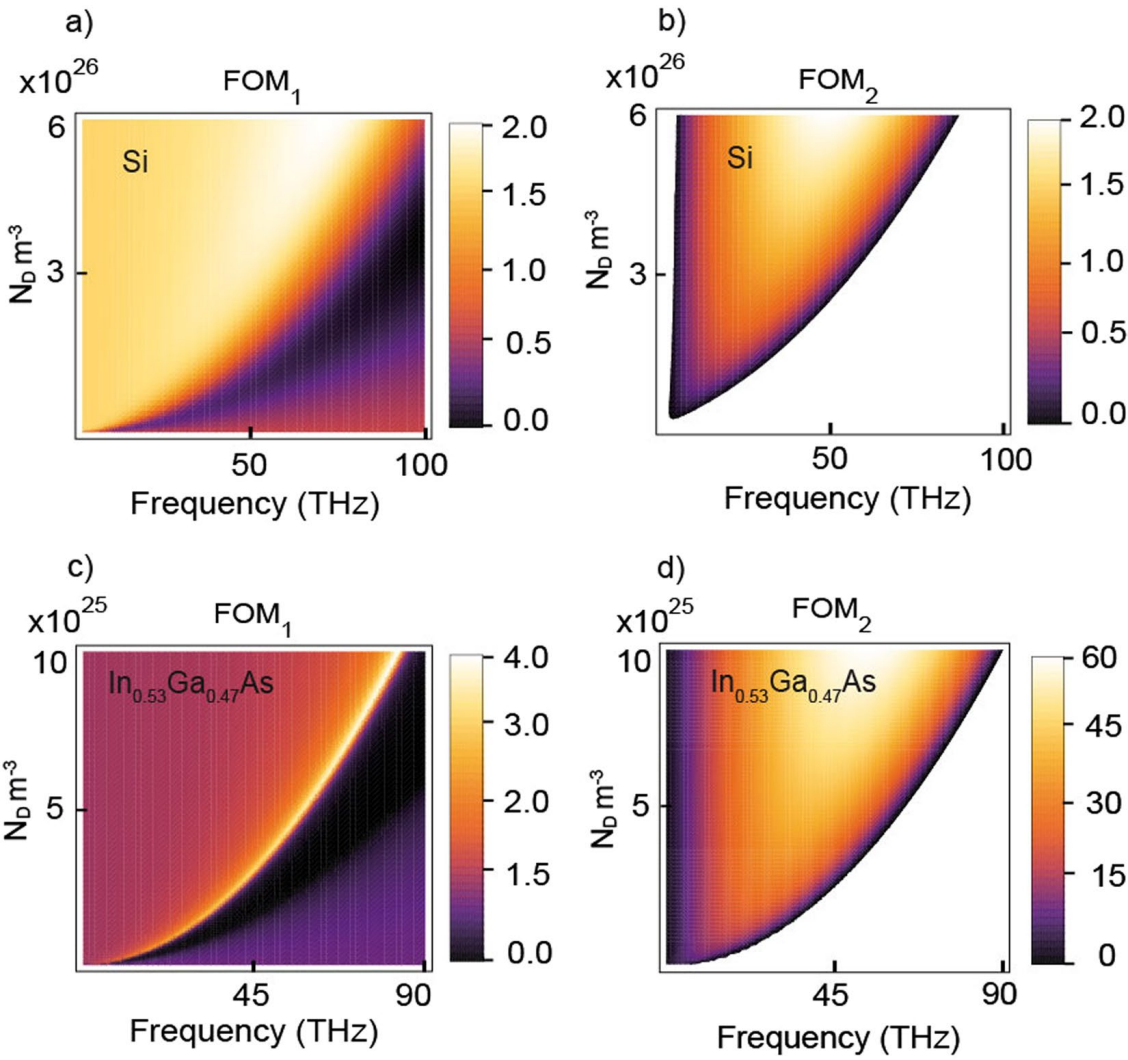
Surface Plasmon Polaritons (SPPs) Figures of Merit (FOM) vs. frequency and doping for (**a**,**b**) Si and (**c**,**d**) In_0.53_Ga_0.47_As.

The FOM_1_ is defined as the ratio of the SPP wavevector *k*_*SPP*_ to the wavevector *k*_*P*_ of bulk waves propagating in the lightly *P*-doped layer (the dielectric layer) and is a measure of how much smaller in size the SPPD can be compared to conventional optical devices. The FOM_2_ describes both the SPPs localization and dissipation losses. An optimal SPPD design is determined by the parametric range where the two figures of merit are large. The FOMs for Si and In_0.53_Ga_0.47_As are calculated for a practically feasible doping and frequency ranges as shown in Fig. 2. As expected our parametric studies show that the SPPD can be formed with minimal physical sizes if the operation frequency is close to the surface plasmon frequency ${\omega }_{sp}=q\sqrt{{N}_{D}/2{\varepsilon }_{p}{\varepsilon }_{0}{m}_{e}}$. The lateral size of the SPPD can be a factor of two (in the case of Si) and a factor of four (in the case of In_0.53_Ga_0.47_As) smaller than that of dielectric devices. However, at the surface plasmon frequency the SPP are highly attenuated. Hence, operation at lower frequencies should be considered as shown by the second figure of merit so that positive trade-off between localization and propagation losses is achieved. The data clearly demonstrates that SPPD based on In_0.53_Ga_0.47_As is expected to manifest both, small device sizes and propagation length that is more than 100 times larger compared to the free space wavelength. In what follows we fix the operation wavelength at 30 THz (corresponding to free space wavelength of 10microns) and adjust the doping concentration accordingly so that an optimal operation is achieved. It must be also noted that in the performed parametric studies we have considered a range of doping concentrations consistent with experimentally attainable values for Si (*N*_*D*_ ≤ 4 × 10^26^ *m* ^−3^)^43^ and In_0.53_Ga_0.47_As (*N*_*D*_ ≤ 8 × 10^25^ *m* ^−3^)^44^.

Since the SPPD switching is due to injection of minority carriers in the presence of an external forward bias voltage, the flow of charge carriers is expected to result in Ohmic heating and corresponding increase of metallurgical junction temperature. The junction temperature is additionally influenced by the electromagnetic energy dissipation of the SPP. To account for these effects we have developed a multi-physics model based on the COMSOL software. The model self-consistently couples the electromagnetic, semiconductor and thermal modules. This is accomplished by developing a MATLAB based facilitator code which shares the inter-dependent physics parameters between the separate modules and allows for self-consistent steady-state and time dependent simulations (see Methods).

**Figure 3:**
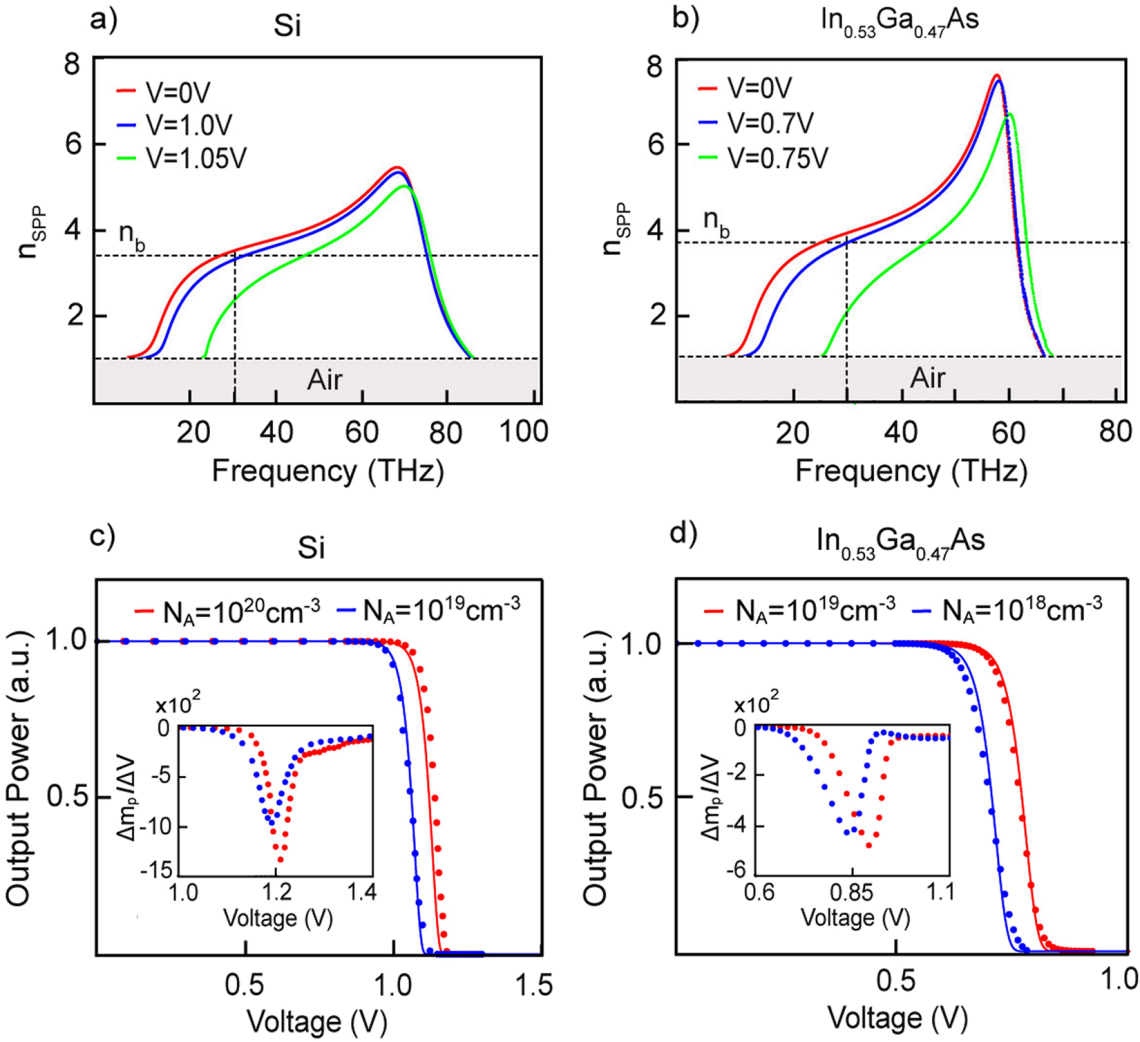
The Surface Plasmon Polaritons (SPPs) dispersion at different external bias voltages for (**a**) Si with doping concentrations;${N}_{A}=1\times {10}^{20}c{m}^{-3},{N}_{D}=4\times {10}^{20}c{m}^{-3}$and (**b**) In_0.53_Ga_0.47_As with ${N}_{A}=1\times {10}^{19}c{m}^{-3},{N}_{D}=5\times {10}^{19}c{m}^{-3}$. The SPPD transmittance for (**c**) Si and (**d**) In_0.53_Ga_0.47_As and different *P*-doping concentrations are obtained using the self-consistent Multiphysics model (dots) and compared to the WKB approximation (solid lines). The corresponding responsivities are shown as inserts. In the calculations the operation frequency is set at 30THz, the thickness of the *P*-type layer is *d* = 1.5 *μm*, *n*_*b*_ identifies the refractive index of *P*- layer and the overall length of the active drift-diffusion region is fixed at *w* = 4 *μm*.

Using the numerical model we begin our study of the SPPD input-output characteristics by first considering the steady state case. The signal modulation of the device is described as the logarithmic ratio of the output/input SPP power densities, *m*_*p*_ = 10 log_10_(*P*_*out*_/*P*_*in*_). As identified above a rapid decrease in power transmission is expected for forward bias larger than the critical *V* > *V*_*c*_, for which the *P*- doped layer acquires metal-like characteristics. Indeed, with increase in the applied voltage the SPP dispersion is rapidly modified as seen in Fig. 3(a,b). For fixed operation frequency *f*_*o*_ = *ω*_*o*_/2*π* = 30 THz and zero applied bias, the SPPs can travel across the device which is exemplified by the fact that the effective refractive index *n*_*SPP*_ of these surface modes is larger than the refractive index *n*_*P*_ of the *P*- doped layer. As the applied voltage approached the critical the SPP dispersion curve is shifted and a refractive index mismatch between SPPs in the drift diffusion region *n*_*SPP*_(*ω*_*o*_, *V*) and in the rest of the device *n*_*SPP*_(*ω*_*o*_, *V* = 0) is observed. This immediately leads to reflection and signal attenuation at the active zone establishing the OFF-state. The actual modulation of the SPP signal is shown in Fig. 3(c,d). Signal modulations surpassing *m*_*p*_ > −100 dB are observed for applied bias above the critical, and responsivities is excess of ${\rm{\Delta }}{m}_{p}/{\rm{\Delta }}V > -1000\,{\rm{dB}}\cdot {{\rm{V}}}^{-1}\,$ for Si and ${\rm{\Delta }}{m}_{p}/{\rm{\Delta }}V > -500\,{\rm{dB}}\cdot {{\rm{V}}}^{-1}$ for In_0.53_Ga_0.47_As are demonstrated (see inserts in Fig. 3(c,d)). Generally increase in acceptor doping concentration (*N*_*A*_) leads to larger responsivity. These results are orders of magnitudes higher compared to the competing optoelectronic devices studied in the literature^8, 9, 32, 45–47^, attesting to the SPPD potential as a high quality switch. The numerical data is also compared to an analytical theory based on the Wentzel-Kramers-Brillouin (WKB) approximation (see Methods), showing remarkable correlation between the two results.

**Figure 4:**
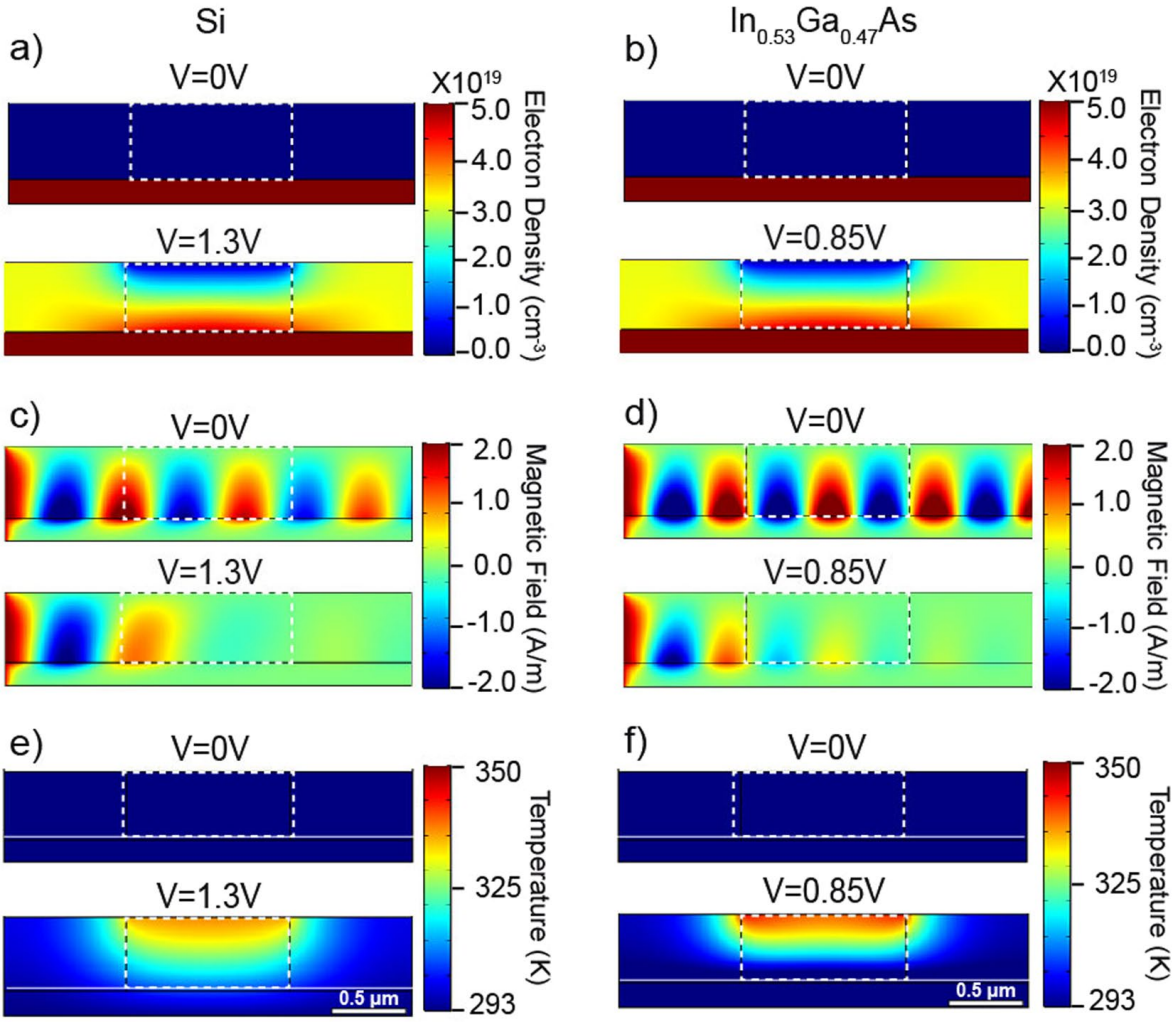
SPPD steady-state response. (**a**,**b**) Steady state minority carrier concentration profiles at different applied voltages. (**c**,**d**) Surface Plasmon Polariton (SPP) propagation along the length of the device. (**e**,**f**) Local temperature profile. In the calculations the operation frequency is set at 30THz, the thickness of the *p*-type layer is *d* = 1.5 *μm* and the overall length of the active drift-diffusion region is fixed at *w* = 4 *μm*.

Figure 4 depicts snapshots of the minority carrier concentration, SPP local field profiles and temperature distributions across the device. For zero external voltage bias the *P*- doped layer is depleted of minority carriers and the SPP modes propagate freely at the *PN* ^+^- junction interface. Close observation of the SPP local field profiles validates the fact that stronger modal localization is achieved for SPPD based on the lattice matched In_0.53_Ga_0.47_As. As the external bias approach/surpass the critical, exponential increase in the minority carrier concentration is observed within the device active region. This results in alteration of the refractive index of the *P*-doped layer leading to reflection and attenuation of SPP modes and the establishment of the OFF-state. Furthermore the flow of charge carriers increases the device temperature due to Ohmic heating; see Fig. 4(e,f). Interestingly, it is evident that minor changes in the electron concentration in close proximity to the metallurgic junction can result in rapid switching of the SPP. This is due to the localized nature of the SPP: these modes are confined to the *PN* ^+^ - junction and slight modification of the electron density near the junction can led to drastic changes in the SPP propagation characteristics as has been already demonstrated in Fig. 3(a,b). Furthermore, the higher local temperatures observed in proximity to the top electrode can be attributed due to two different processes. First, the local current density achieves maximum values near the anode (which acts as a sink) and second the integrated convective heat transfer is higher at the position of the cathode (predominantly due to larger surface area). From the data it is also evident that the In_0.53_Ga_0.47_As device switches at lower applied voltages as compared to Si device. This is due to the fact that the critical voltage *V*_*c*_ increases logarithmically with electron effective mass which for Si is six times higher than for In_0.53_Ga_0.47_As.

**Figure 5:**
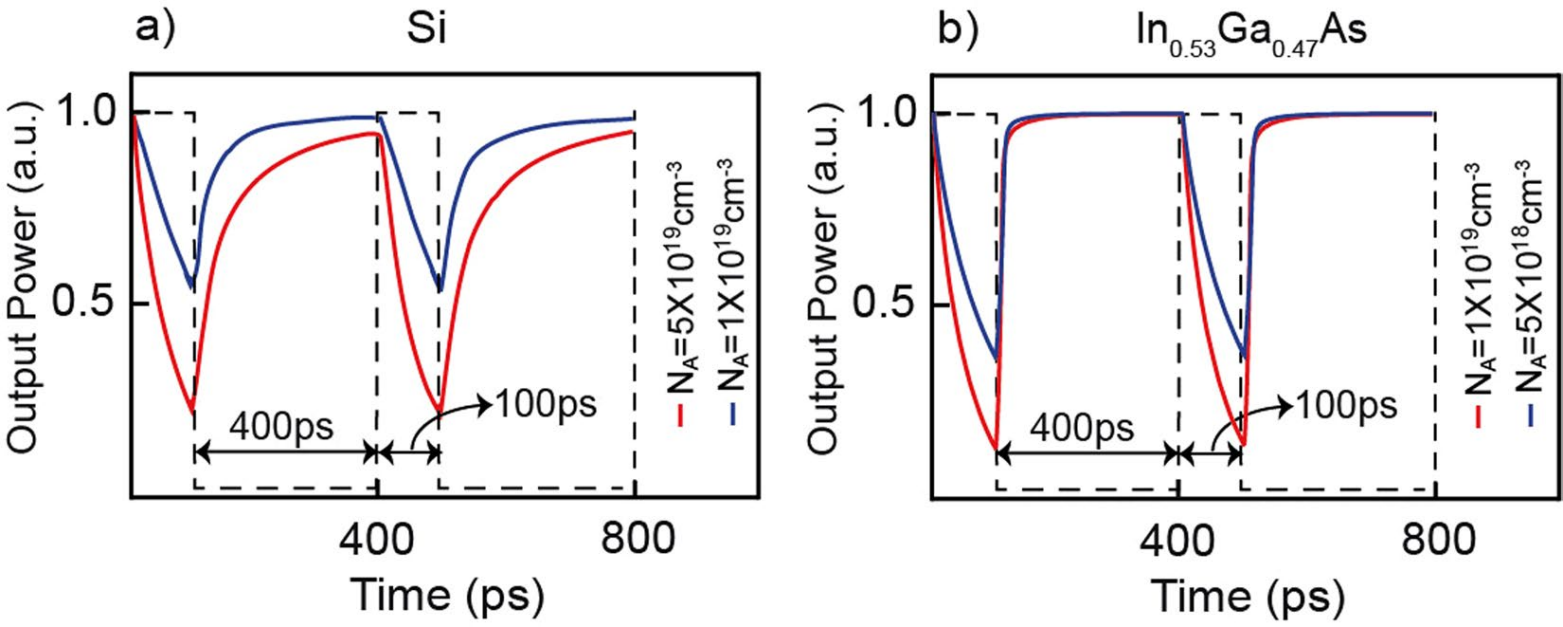
SPPD switching. SPPD output signal (solid line) under step-type of input voltage (dashed black line) with magnitude (**a**) 1.3 V for Si and (**b**) 0.9 V for In_0.53_Ga_0.47_As. The signal is repetitively switched following the external voltage. In the calculations the operation frequency is set at 30 THz, and the donor doping concentration is ${N}_{D}=4\times {10}^{20}c{m}^{-3}$ for the Si device and ${N}_{D}=5\times {10}^{19}c{m}^{-3}$ for the In_0.53_Ga_0.47_As device.

A major interest in plasmonic based optoelectronic switching can be attributed to their perceived fast temporal response^8, 25, 34–36^. To assess the response times of the SPPD we have performed a transient analyses under step type input voltage bias. Our results for sets of acceptor concentrations are shown in Fig. 5. In the calculations the maximum values of the input voltages are fixed above the corresponding critical values *V*_*c*_ = 1.14 *V* for Si and *V*_*c*_ = 0.81 *V* for In_0.53_Ga_0.47_As. The obtained results show a well-defined distinction between the OFF and ON rise times, with the former being substantially faster. This distinction can be attributed to the drastically different physical processes that are involved, with the OFF times governed by the electric field facilitated injection of minority carriers in the *P*- layer while the ON times are set by charge diffusion (see Methods). Consistently, a faster response times are observed for the In_0.53_Ga_0.47_As device. Moreover, the modulation rates are revealed to dependent on the acceptor doping concentration (*N*_*A*_), with higher doping leading to faster signal modulation rates.

**Figure 6:**
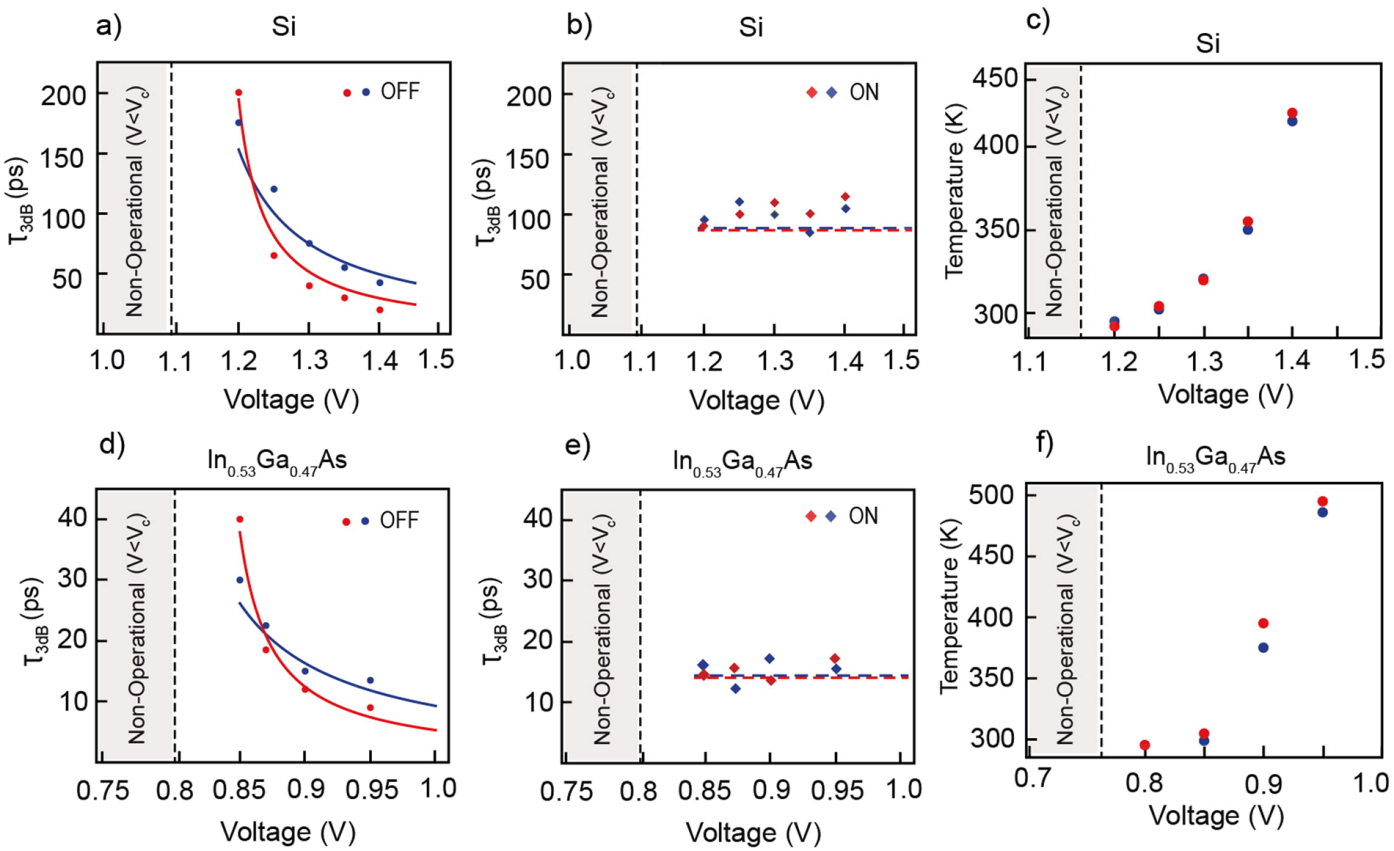
SPPD response times and operation temperatures. (**a**,**d**) Dependence of the SPPD OFF times on the applied voltage (*V* > *V*_*c*_). The device OFF time is dependent on the electron drift into the *P*-region and is thus inversely proportional to the applied voltage. (**b**,**e**) Dependence of the SPPD ON times on the applied voltage (*V* > *V*_*c*_). (**c**,**f**) The maximum temperature recorded inside the device vs. the applied voltage. In the calculations the operation frequency is set at 30 THz. The doping concentrations for the Si device are ${N}_{A}=1\times {10}^{19}c{m}^{-3}$ (blue dots) and ${N}_{A}=1\times {10}^{20}c{m}^{-3}$ (red dots) with $\,{N}_{D}=4\times {10}^{20}c{m}^{-3}$, whereas for the In_0.53_Ga_0.47_As device the doping concentrations are ${N}_{A}=1\times {10}^{19}c{m}^{-3}$ (red dots) and ${N}_{A}=1\times {10}^{18}c{m}^{-3}$ (blue dots) with $\,{N}_{D}=5\times {10}^{19}c{m}^{-3}$. The doping influences both the width of the space charge region and the electron mobility, thus modifies to a different extent the ON and OFF times. In the figures the numerical data (dots diamonds) is compared to the analytical drift-diffusion model (solid lines).

To assess the true potential of the SPPD as a fast optoelectronic switch we have performed extensive parametric analyses of the 3 dB ON (${\tau }_{3dB}^{ON}$)/OFF (${\tau }_{3dB}^{OFF}$) times and maximum local temperature at the drift-diffusion region. The response times and temperature for different applied voltages and doping concentrations are shown is Fig. 6. The OFF times are found to be inversely proportional on the applied voltage while the ON times are near independent on the voltage. Again this behavior can be explained using a simple drift-diffusion model of the election concentration advance through the active region (see Methods). The SPPD transient response is dependent on the acceptor doping due to changes in the minority carrier mobility. Since the electron mobility of In_0.53_Ga_0.47_As is higher compared to Si we obtain faster response times for the former. For sufficiently high applied voltages, 3 dB data rates in excess of 50Gbit/s can be obtained for In_0.53_Ga_0.47_As while moderate rates of up to 10Gbit/s are expected for Si. However, the increase in the applied voltage can lead to increase in heat dissipation. This is clearly visible in Fig. 6(c,d) where the maximum local temperatures within the device are depicted. To minimize heating for the considered device sizes the applied forward bias should not exceed 1.4 V in the case of Si and 1 V in the case of In_0.53_Ga_0.47_As. Further minimization of the power dissipation can be accomplished by reducing the size of the SPPD drift-diffusion region and varying the *N*- layer doping while keeping the SPP operation frequency within the experimentally accessible mid-IR spectral range.

**Figure 7:**
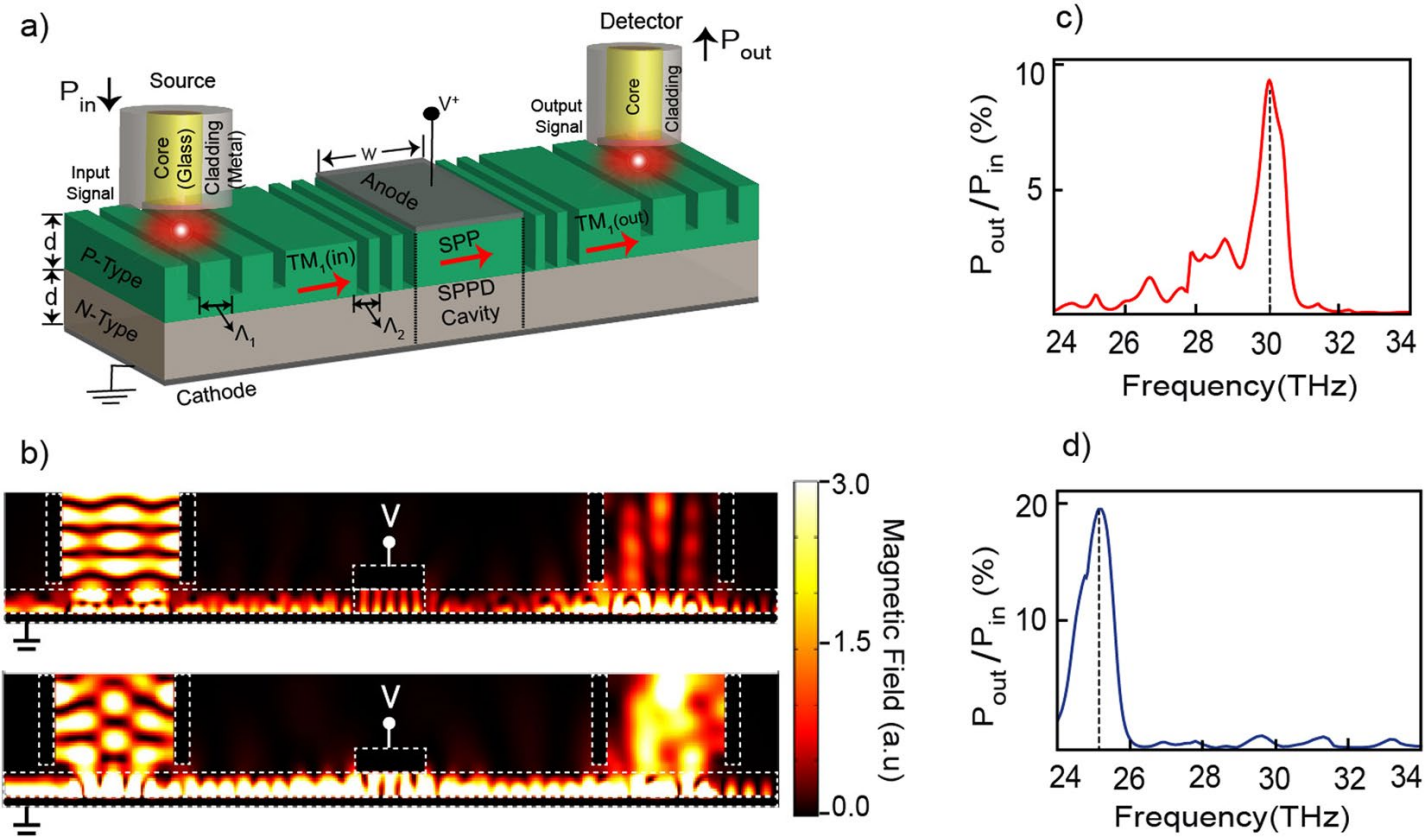
SPPD coupling/decoupling scheme. (**a**) Schematic of the SPPD with input and output waveguides coupling. (**b**) Magnetic field intensity for two different input grating ${{\Lambda }}_{1}=6\,\mu m$ (top) and ${{\Lambda }}_{1}=5\,\mu m$ (bottom) at constant SPP cavity grating ${{\Lambda }}_{2}=2.6\,\mu m$. (**c**) SPP coupling efficiency for the ${{\Lambda }}_{1}=6\,\mu m$ (top) grating showing optimized efficiency at operating frequency of 30 THz. (**d**) SPP coupling efficiency for the ${{\Lambda }}_{1}=5\,\mu m$ (bottom) grating showing optimized efficiency at operating frequency of 25 THz.

Finally, for the experimental implementation and characterization of the SPPD it is crucial to introduce an efficient coupling/decoupling scheme. In what follows we consider local SPP excitation and detection using a pair of far–infrared single mode fibers and set of gratings. The overall geometry of the proposed set up is shown in Fig. 7a. The input radiation is coupled first to a low loss dielectric-dielectric-metal (DDM) waveguide mode of the *P*-layer, either TM1 for planar device or TM11 for rectangular device, which are then fed into a SPPD cavity via an etched grating. The cavity length is set at 7 μm to support low order SPP resonances with a cavity height of 1.8 μm. The SPPD output signal is then fed into the output fiber with a second grating. All simulations are performed using the self-consistent optoelectronic-model as specified above. The local magnetic field profile for the planar device is shown in Fig. 7b. The excitation of the lower wavelength SPP cavity mode is clearly visible within the drift-diffusion region. In the calculations we have used two set of input grating *Λ*_1_ = 6 μm (top) and *Λ*_1_ = 5 μm (bottom) with a fixed output grating of *Λ*_2_ = 2.6 μm. The grating periods have been optimized to obtain better coupling/decoupling efficiencies at two operational frequencies; 30 THz (top) and 25 THz (bottom). This is shown in Fig. 7(c,d) where the output power efficiency is as high as ~9% (for 30 THz) and up to ~20% (for 25 THz). The efficiency is calculated as the ratio *P*_*out*_/*P*_*in*_, where *P*_*out*_ is the power density at the output fiber and *P*_*in*_ is the power flow through the input fiber. It must be noted that due to the used symmetric input grating the decoupling efficiencies are fundamentally restricted to less than 50%. Alternatively, asymmetric grating configurations could also be considered to further improve the collected output signal. Regardless, the demonstrated efficiencies are sufficient for the practical demonstration of the device.

In conclusion we have demonstrated Surface Plasmon Polariton Diode (SPPD) based on degenerated Si and lattice matched In_0.53_Ga_0.47_As *PN* ^+^- junctions. Multiphysics numerical code was developed to self-consistently model the electromagnetic, solid state and for a first time thermal response of the device. Our numerical analysis suggests that a SPPD based on lattice matched In_0.53_Ga_0.47_As can operate at responsivities in excess of −600 dB·V^−1^ and data rates up to 50 Gbit/s. Moreover, owning to the use of surface plasmon polaritons the proposed optoelectronic switch can have physical dimensions substantially smaller compared to conventional optical devices. Finally, a practical design based on a set of coupling/out-coupling gratings is proposed for the experimental demonstration of the optoelectronic switch.

## Methods

### Surface Plasmon Polaritions at degenerate semiconductor junctions

Similar to metals, the optical response of highly doped degenerate semiconductors, can be characterized by the Drude model with permittivity given as $\varepsilon (\omega )={\varepsilon }_{b}-{\omega }_{p}^{2}(n,T)/({\omega }^{2}+i\omega {\omega }_{\tau }(n))\,$, where the bound electron permittivities are *ε*_*b*_ = 11.6 for Si and 13.9 for In_0.53_Ga_0.47_As^48^. An important difference to note is that both the plasma frequency, ${\omega }_{p}=q\sqrt{n(V,T)/{\varepsilon }_{0}{m}_{e}}$, and relaxation rate, ${\omega }_{\tau }=e/{m}_{e}\mu ({N}_{A},{N}_{D},T)$, now depend on the doping concentrations and local temperature. This dependence allows for fine tuning of the optical properties of the doped semiconductors at far-infrared and THz frequencies. For the three layer Surface Plasmon Polariton Diode (SPPD) (see Fig. 1) that we considered in this work, the dispersion relationship of the surface modes guided along the semiconductor *PN* ^+^- junction is given by^49^

2 $${e}^{-4{k}_{p}d}=(\frac{{k}_{p}/{\varepsilon }_{p}+{k}_{n}/{\varepsilon }_{n}}{{k}_{p}/{\varepsilon }_{p}-{k}_{n}/{\varepsilon }_{n}})\,(\frac{{k}_{p}/{\varepsilon }_{p}+{k}_{a}/{\varepsilon }_{a}}{{k}_{p}/{\varepsilon }_{p}-{k}_{a}/{\varepsilon }_{a}})$$

where $\,{\varepsilon }_{n}$, ${\varepsilon }_{p}$, ${\varepsilon }_{a}$ are the dielectric permittivity’s of *N*- doped layer, *P*-doped layer and surrounding dielectric (air), respectively, *d* is the thickness of the *P*- layer, and ${k}_{l}=\sqrt{{k}_{spp}^{2}-{\varepsilon }_{l}{k}_{0}^{2}}$ are the transversal SPP wavevectors in the three layers $l\in \,\{n,p,a\}$. Equation 2 can be used to qualitative and quantitative understand the switching of the SPP pertaining to the SPPD. For this we apply the Wentzel-Kramers-Brillouin (WKB) method^50^ which gives the SPP transmittance across the device as, ${P}_{out}/{P}_{in}\approx {|\exp (-{\rm{Im}}{\int }_{0}^{w}{k}_{spp}(x,V)dx)|}^{2}$, where *w* is the width of the active drift-diffusion region. For input voltages much larger than the critical, we have ${k}_{spp}\approx {k}_{0}\sqrt{\,{\varepsilon }_{n}/2}\approx i\frac{{\omega }_{p}}{c\sqrt{2}}$, and the SPP transmission across the device active zone is significantly impeded as $\,{P}_{out}/{P}_{in}\approx \exp (-\sqrt{2}\frac{{\omega }_{p}w}{c})\ll 1$, where ${\omega }_{p}=q\sqrt{{N}_{D}/{\varepsilon }_{0}{m}_{e}}$ is the plasma frequency of the *N*- layer.

### Response times

A simple analytical expressions of the SPPD response times can be obtained from the drift-diffusion equations. The OFF-times are governed by the electric field facilitated drift of minority carriers across the active zone. Since the SPPs are strongly localized at the *PN* ^+^ depletion region we can write the response times as $\,{\tau }_{{\rm{OFF}}}={l}_{spp}/{v}_{d}$, where ${l}_{spp}=1/{k}_{z}^{spp}=1/2\sqrt{{k}_{spp}^{2}-{\varepsilon }_{p}{k}_{0}^{2}}$is the SPP field penetration depth in the *P*- layer and ${v}_{d}\approx {\mu }_{e}^{p}(V-{V}_{c})/{x}_{p}$ is the drift velocity, where ${\mu }_{e}^{p}$ is the minority carrier drift mobility^51, 52^ and *x*_*p*_ is the thickness of the *P*-layer. When the applied voltage is removed the minority carriers ON times are diffusion limited and given as $\,{\tau }_{{\rm{ON}}}={l}_{spp}^{2}/(2{D}_{n})$, where ${D}_{n}=(kT/q){\mu }_{e}^{n}$ is the diffusion co-efficient of the electrons in the *P*- layer^43, 44^.

### Numerical Model

To have a proper understanding of the complex Multiphysics processes governing the SPPD operation a self-consistent thermo-electro-optic model is developed. A finite difference integrated circuits COMSOL Semiconductor Module (CSM) is implemented to obtain the minority carriers distribution across the device, which is then used to extract the inhomogeneous dielectric function of the *P*- layer. Full-wave finite difference (FD) calculations of the SPPD electromagnetic response are then performed using the COMSOL Electromagnetic Module (CEM). As discussed in the main text of this article the SPPD response is also sensitive to the thermal effects due to Ohmic heating which are accounted for by the COMSOL Heat Transfer Module (CHTM). A seamless integration between the three physical modules is facilitated through a common Matlab graphical user interface (GUI). The response times are estimated by fitting the transmittance data with an exponential function *e* ^−*t*/*τ*^, where *τ* is the rise/fall time. The 3 dB response times are then estimated using the standard procedure $10{\mathrm{log}}_{10}({e}^{-\frac{{\tau }_{3{\rm{dB}}}}{\tau }})=-3$.
